# Acupuncture for chronic fatigue syndrome and idiopathic chronic fatigue: a multicenter, nonblinded, randomized controlled trial

**DOI:** 10.1186/s13063-015-0857-0

**Published:** 2015-07-26

**Authors:** Jung-Eun Kim, Byung-Kwan Seo, Jin-Bong Choi, Hyeong-Jun Kim, Tae-Hun Kim, Min-Hee Lee, Kyung-Won Kang, Joo-Hee Kim, Kyung-Min Shin, Seunghoon Lee, So-Young Jung, Ae-Ran Kim, Mi-Suk Shin, Hee-Jung Jung, Hyo-Ju Park, Sung-Phil Kim, Yong-Hyeon Baek, Kwon-Eui Hong, Sun-Mi Choi

**Affiliations:** Acupuncture, Moxibustion & Meridian Research Group, Korea Institute of Oriental Medicine, Daejeon, South Korea; Department of Acupuncture & Moxibustion, Kyung Hee University Hospital at Gangdong, Seoul, South Korea; Department of Oriental Rehabilitation Medicine, Gwangju Oriental Hospital of Dongshin University, Gwangju, South Korea; Department of Oriental Gynecology, Jecheon Oriental Hospital of Semyung University, Jecheon, South Korea; College of Korean Medicine, Gachon University, Seongnam, South Korea; Department of Acupuncture & Moxibustion, Kyung Hee University Medical Center, Seoul, South Korea; Department of Acupuncture & Moxibustion, Daejeon Oriental Hospital of Daejeon University, Daejeon, South Korea; Department of Korean Medicine, Nurije Korean Medical Clinic, Daejeon, South Korea

**Keywords:** acupuncture, fatigue, randomized controlled trials as topic

## Abstract

**Background:**

The causes of chronic fatigue syndrome (CFS) and idiopathic chronic fatigue (ICF) are not clearly known, and there are no definitive treatments for them. Therefore, patients with CFS and ICF are interested in Oriental medicine or complementary and alternative medicine. For this reason, the effectiveness of complementary and alternative treatments should be verified. We investigated the effectiveness of two forms of acupuncture added to usual care for CFS and ICF compared to usual care alone.

**Methods:**

A three-arm parallel, non-blinded, randomized controlled trial was performed in four hospitals. We divided 150 participants into treatment and control groups at the same ratio. The treatment groups (Group A, body acupuncture; Group B, Sa-am acupuncture) received 10 sessions for 4 weeks. The control group (Group C) continued usual care alone. The primary outcome was the Fatigue Severity Scale (FSS) at 5 weeks after randomization. Secondary outcomes were the FSS at 13 weeks and a short form of the Stress Response Inventory (SRI), the Beck Depression Inventory (BDI), the Numeric Rating Scale (NRS), and the EuroQol-5 Dimension (EQ-5D) at 5 and 13 weeks.

**Results:**

Group A showed significantly lower FSS scores than Group C at 5 weeks (*P* = 0.023). SRI scores were significantly lower in the treatment groups than in the control group at 5 (Group A, *P* = 0.032; B, *P* <0.001) and 13 weeks (Group A, *P* = 0.037; B, *P* <0.001). Group B showed significantly lower BDI scores than Group C at 13 weeks (*P* = 0.007). NRS scores from the treatment groups were significantly reduced compared to control at 5 (Group A and B, *P* <0.001) and 13 weeks (Group A, *P* = 0.011; B, *P* = 0.002).

**Conclusions:**

Body acupuncture for 4 weeks in addition to usual care may help improve fatigue in CFS and ICF patients.

**Trial registration:**

Clinical Research Information Service (CRIS) KCT0000508; Registered on 12 August 2012.

**Electronic supplementary material:**

The online version of this article (doi:10.1186/s13063-015-0857-0) contains supplementary material, which is available to authorized users.

## Background

Fatigue generally refers to severe exhaustion during and after daily activities or to a lack of energy. Prolonged and repetitive fatigue seriously influences the quality of life [1]. Fatigue that continues for more than 6 months is defined as chronic fatigue, and chronic fatigue for which no medical explanation exists is classified as chronic fatigue syndrome (CFS) or idiopathic chronic fatigue (ICF). The diagnostic criteria for CFS are as follows: (1) the individual has severe chronic fatigue for 6 or more consecutive months that is not due to ongoing exertion or other medical conditions associated with fatigue; (2) the fatigue significantly interferes with daily activities and work; and (3) the individual concurrently has four or more of eight specific symptoms. These symptoms include the following: (i) postexertional malaise lasting for more than 24 h; (ii) unrefreshing sleep; (iii) significant impairment of short-term memory or concentration; (iv) muscle pain; (v) multijoint pain without swelling or redness; (vi) headaches of a new type, pattern, or severity; (vii) tender cervical or axillary lymph nodes; and (viii) a sore throat that is frequent or recurring. Information about CFS can be found at http://www.cdc.gov/cfs/case-definition/index.html. Cases that cannot be categorized according to the above diagnostic criteria are included under ICF [2, 3]. The worldwide prevalence of ICF and CFS is approximately 10 % and 1 %, respectively [4]. The causes of CFS and ICF are not clearly known. Immune regulatory dysfunction, various inflammatory conditions, neuroendocrine dysfunction, central nervous system impairments, and stress are the hypothesized causes of them. There is no definitive cure for CFS and ICF. Therefore, patients with CFS and ICF are interested in Oriental medicine or complementary and alternative medicine, and various alternative approaches have been used to treat these patients [3, 5, 6]. For this reason, the effectiveness of complementary and alternative treatments should be verified.

Acupuncture and moxibustion are among the most widely used treatments in Oriental medicine. Thus far, some systematic reviews have reported favorable outcomes regarding the effects of acupuncture and moxibustion on CFS. However, the overall finding of these reviews is that firm evidence for the effects of acupuncture and moxibustion is lacking because of the poor methodological quality of the studies included [7–11]. In this trial, we evaluated the effectiveness of two acupuncture methods: (1) body acupuncture, a traditional acupuncture method widely used for many years in China, Korea, and Japan, in which treatment is applied to the whole body, and (2) Sa-am acupuncture, a Korean traditional acupuncture method developed by Sa-am in the Chosun Dynasty, in which the five-phase theory and mother-child reinforcement-reduction principle are applied to the selection of points and needling manipulation [12–14]. This study aimed to evaluate the overall effects of acupuncture treatment by comparing outcomes in a treatment group receiving body acupuncture or Sa-am acupuncture with outcomes in a control group receiving usual care.

## Methods

This was a three-arm, randomized, nonblinded, controlled, and parallel-designed trial. This trial was conducted in four clinical research centers of South Korea from August 2012 to May 2013. The participants were recruited by each center (Gwangju, Seoul, Jecheon, and Daejeon) through advertisements in local newspapers, the websites of local universities, and posters displayed in hospitals. Details of the study design are available in a previous publication [15]. The flow diagram and CONSORT (Consolidated Standards of Reporting Trials) checklist are provided in Additional files 1 and 2.

### Ethical considerations

This study was approved by the institutional review boards of the Gwangju Oriental Hospital of Dongshin University, Kyung Hee University Hospital at Gangdong, Jecheon Oriental Hospital of Semyung University, and Daejeon Oriental Hospital of Daejeon University. All participants provided written informed consent. The study was conducted according to the principles of the Declaration of Helsinki [16].

### Inclusion criteria

The inclusion criteria for the study were as follows [2, 17]:

1. Adult men and women aged 19 to 65 years.
2. Presenting major symptoms of continuous or recurrent fatigue due to unknown causes of at least 6 months’ duration.
3. Absence of abnormal findings in the following: blood pressure (BP) test, blood tests (hemoglobin (Hb), hematocrit (Hct), white blood cells (WBC), and glucose), biochemical marker tests (aspartate aminotransferase (AST), alanine aminotransferase (ALT), and creatinine), serum electrolyte tests (sodium (Na), potassium (K), and chloride (Cl)), thyroid function tests (TFTs) (thyroid stimulating hormone (TSH) and free thyroxine (FT4)), pregnancy test for women of child-bearing age, chest radiography (CXR), and electrocardiography (ECG).

Test findings were considered “abnormal” with reference to the following values:

- BP: Diastolic BP ≥90 mmHg (average of 2 measurements, each taken at an interval of at least 2 min after a rest period of at least 5 min in a sitting position).
- Blood tests: Hb <13 or <11.5 g/dL and Hct <38 % or <36 %, for males and non-pregnant females, respectively; WBC ≥11,000/mm^3^ and random plasma glucose ≥200 mg/dL for all participants.
- Biochemical marker tests: AST or ALT ≥50 IU/L; creatinine ≥1.5 mg/dL.
- Electrolyte tests: Na <135 or ≥145 mmol/L; K <3.5 or ≥5.5 mmol/L; Cl <97 or ≥110 mmol/L.
- TFTs: TSH <0.35 or ≥5.50 mIU/mL; FT4 <0.89 or ≥1.76 ng/dL.
- Pregnancy test: Urine sample positive for human chorionic gonadotropin (hCG).
- CXR: Active pulmonary tuberculous lesions.
- ECG: Indications of heart disease requiring treatment, such as arrhythmia, ischemic heart disease, or cardiomegaly.

4. Numeric Rating Scale (NRS) [18] score ≥4 during the 7 days preceding the screening tests.
5. Providing written informed consent after being informed of the objectives and particularities of the clinical trial, and agreeing to participate in the trial.

### Exclusion criteria

The exclusion criteria for the study were as follows [2, 17]:

1. History of chronic fatigue or a present illness that might trigger chronic fatigue, including (but not limited to) the following disorders:

- Organic causes: Acute or chronic liver diseases (hepatitis, liver cirrhosis), anemia, tuberculosis, chronic lung diseases, cardiovascular diseases (heart failure, hypertension), endocrine/metabolic diseases (diabetes, hyperthyroidism, hypothyroidism, severe obesity with a body mass index (BMI) of ≥35 kg/m^2^), autoimmune diseases (rheumatoid arthritis, systemic lupus erythematosus, multiple sclerosis), malignant tumors, or infectious diseases.
- Psychosocial causes: Major depression, anxiety neurosis, a recent severe stress, schizophrenia, alcoholism, or an eating disorder (anorexia nervosa, bulimia nervosa).

2. Medication with the following drugs during the preceding 2 weeks: Antihypertensive drugs, antidepressants, antianxiety agents, sleeping pills, or antihistamines.
3. Female participants who were pregnant, breast-feeding, or planning for pregnancy.
4. Participating in other clinical trials.
5. Undertaking excessive workloads (for example, undertaking multiple jobs).
6. History of hypersensitivity reaction to acupuncture treatment.
7. Residents of collective dwelling facilities, such as social welfare institutions.
8. Failure to provide written informed consent.
9. Considered unfit for the trial by the principal investigator because of other reasons.

### Randomization

A statistician used a computer program (Strategic Applications Software (SAS), version 9.1.3 (SAS Institute Inc., Cary, NC, USA) to generate the random allocation sequence. We used a stratified block randomization scheme with four institutions chosen as strata. The generated sequences were sealed in opaque envelopes and delivered to each center, where they were stored in double-locked cabinets. Randomization was conducted for participants who satisfied all selection criteria. The researcher opened the random allocation envelopes in order in front of the participants and assigned them to 1 of 3 groups. The clinical research coordinator allotted participant identification codes and recorded them in the case report forms. The opened envelopes were stored separately in double-locked cabinets.

### Blinding

This was a nonblinded study.

### Interventions

The eligible participants were allocated randomly into three groups, including Group A, Group B, or Group C. In each group, participants received their allocated treatment. Participants in treatment group received 10 sessions of acupuncture treatment for 4 weeks, 2 to 3 times a week, using stainless steel disposable needles (0.25-mm diameter, 30-mm length; Dongbang Acupuncture Inc., South Korea). Participants were informed about the body acupuncture and Sa-am acupuncture used in this study as follows: “In this study, two types of acupuncture will be used. One type will be body acupuncture, which is based on traditional Chinese medicine (TCM). The other type will be Sa-am acupuncture, a traditional Korean acupuncture method.” The practitioners, doctors of Korean medicine having more than 3 years of practical experience, participated in a training session to ensure that treatments were administered consistently. The practitioners were allowed to communicate with the participants about treatment, lifestyle, etc.

### Group A (body acupuncture plus usual care group)

The participants in Group A were treated in the supine position at the acupoints described below. The acupoints were selected based on published literature and textbooks [12, 19]:

1. GV20 (forward horizontal needling up to a depth of 0.5 to 1.5 cun).
2. Bilateral GB20 (perpendicular needling toward the opposite-side eye up to a depth of 0.3 to 1.0 cun).
3. Bilateral BL11, BL13, BL15, BL18, BL20, and BL23 (downward oblique or horizontal needling up to a depth of 0.5 to 1.0 cun).

All needles were retained for 15 min without twirling. In TCM, the *qi* moves through the meridians at a rate of 6 cun per respiratory cycle. The length of the meridians and collaterals equals 1620 cun. Thus, 270 (1620/6) respiratory cycles are required for *qi* to make one cycle through the body. If an adult breathes 18 times per min, 15 min (270/18) are needed for *qi* to make one cycle [20]. Therefore, the needle retaining time was 15 min.

### Group B (Sa-am acupuncture plus usual care group)

The participants assigned to Group B received treatment at the following acupoints while sitting on a chair. The acupoints were selected based on a published literature [13]:

1. LU8 (downward oblique needling up to a depth of 0.5 to 0.7 cun)
2. SP3 (upward oblique needling up to a depth of 0.3 to 0.5 cun)
3. HT8 (downward oblique needling up to a depth of 0.3 to 0.5 cun)
4. BL15 (upward oblique or horizontal needling up to a depth of 0.5 to 1.0 cun)
5. CV6 (downward oblique needling up to a depth of 0.5 to 1.0 cun).

All needles were retained for 15 min after being twirled nine times (at LU8, SP3, and HT8) or six times (at BL15 and CV6).

### Group C (usual care alone group)

The participants randomized to Group C did not receive acupuncture treatment; however, they did receive the necessary usual care [21, 22].

### Concomitant treatments

In all groups, usual care consisted of the use of any form of concomitant treatment, including Oriental medicine treatments (for example, acupuncture for conditions other than chronic fatigue, moxibustion, herbal medicine), Western medicine (for example, conventional medication, injections), or self-care (for example, dietary supplements, exercise). All groups were provided with educational materials about chronic fatigue.

### Primary outcome

The Fatigue Severity Scale (FSS) score at 5 weeks after randomization was used as the primary endpoint. The FSS included nine questions, scored on a scale of 1 to 7, which were used to evaluate the participant’s fatigue level during the previous week [23, 24].

### Secondary outcomes

A short form of the Stress Response Inventory (SRI), consisting of 22 questions covering the following three categories, was used to evaluate stress: somatization (nine items), depression (eight items), and anger (five items) [25]. The Beck Depression Inventory (BDI) consisted of 21 questions assessing the cognitive, emotional, motivational, and somatic symptoms of depression. Each item was scored on a 4-point scale from 0 to 3 [26]. The NRS used a horizontal straight line with numbers from 0 to 10 and indications of “no fatigue” on the far left and “most severe fatigue experienced” on the far right end. The participants were asked to select the number that represented the level of their own fatigue [18]. The EuroQol-5 Dimension (EQ-5D) tool assessed health-related quality of life. It consisted of the EQ-Visual Analogue Scale (VAS), which scored the participant’s health condition on a straight line marked from 0 to 100, and the EQ-5D descriptive system, which inquired about five dimensions of the participant’s current health status. Information about the EQ-5D can be found at http://www.euroqol.org/.

### Other assessments

We assessed the treatment expectancy survey [27] completed by each participant, and confirmed that the Centers for Disease Control and Prevention (CDC)’s diagnostic criteria for CFS [2] was fulfilled.

### Adverse events

Practitioners recorded all unexpected and unintended responses as reported by each participant on an adverse event report form. The relationship between acupuncture treatment and adverse events was graded from 1 to 6 (1 = definitely related, 2 = probably related, 3 = possibly related, 4 = probably not related, 5 = definitely not related, and 6 = unknown).

### Statistical analysis

In order to have a 90 % chance of detecting a mean difference of 0.915 in the primary outcome at a significance level of 5 %, 40 patients were required per group. Based on the effect size described in a previous pilot study [12], we assumed a mean change of 0.9632 in the treatment group and 0.0482 in the control group, with a common standard deviation of 1.1479. Allowing for a 20 % dropout rate, 50 patients were required per group (150 in total). Details of the sample size calculation are available in a previous publication [15]. A computer program (SAS, version 9.1.3) was used to perform the analysis. The hypothesis of this study was as follows: *H*_0_ : *μ*_1_ = *μ*_3_, *μ*_2_ = *μ*_3_, *H*_1_ : *not H*_0_ (*μ*_1_, FSS mean score for Group A at 5 weeks after randomization; *μ*_2_, FSS mean score for Group B at 5 weeks after randomization; and *μ*_3_, FSS mean score for Group C at 5 weeks after randomization). The analysis set consisted of a full analysis set (FAS; that is, the set of participants who were as close as possible to the intention-to-treat (ITT) principle) and a per-protocol set (PPS; that is, the set of participants who were more compliant with the protocol) [28]. The FAS was used for the main analysis.

A mixed model for repeated measures (MMRM) method was used to determine the differences between the experimental groups and the control group; the treatment, visit time, and institution were the fixed factors, and the participant was the random factor [29]. Additionally, an analysis of covariance (ANCOVA) was performed by replacing the missing data using the last observation carried forward (LOCF) method, and the results were compared with the results of the MMRM method. In ANCOVA, the primary and secondary outcomes at 5 and 13 weeks after randomization were the dependent variables, the baseline value was the covariate, and the group and the institution were the fixed factors. The analyses were performed at the 5 % significance level. When the ANCOVA produced significant results, a post-hoc analysis was conducted to determine which groups showed differences. For the socio-demographic characteristics and treatment expectancy, the mean and standard deviation were determined from the continuous data, and the frequency and percentage were determined from the categorical data. The subgroup analysis of FSS was performed in CFS and ICF groups.

## Results

### Flow of participants

We selected 150 participants from 195 screened individuals. Gwangju, Kyung Hee, and Jecheon hospitals recruited 45 participants each, and Daejeon center recruited 15 participants. The selected participants were randomly allocated to a treatment group (Group A or B) or the control group (Group C). Among the participants, three from Group A, two from Group B, and two from Group C dropped out of the study (see Additional file 1). The reasons the participants dropped out of the study are presented in Additional file 1.

### Baseline characteristics

The sociodemographic characteristics of the participants are shown in Table 1. No significant differences were observed between the groups regarding age, sex, height, weight, education level, occupation, marital status, meal regularity, exercise, smoking, and use of alcohol. According to the CFS questionnaire, 63 participants (42 %) could be classified as having CFS. The frequency of participants with CFS was similar within the different groups. The treatment expectancy was significantly different between the treatment groups and the control group (Table 1).

**Table 1:** Baseline characteristics of study participants

Variable		Group A (n = 49)	Group B (n = 51)	Group C (n = 50)
Age, years		44.9 ± 11.4	39.6 ± 11.5	41.1 ± 11.9
Sex, M/F		15 (30.6)/34 (69.4)	18 (35.3)/33 (64.7)	19 (38.0)/31 (62.0)
Height, cm		162.2 ± 6.8	165.3 ± 8.9	164.4 ± 8.8
Weight, kg		59.8 ± 10.8	63.0 ± 12.5	62.8 ± 12.4
Duration of chronic fatigue				
	6 months to 1 year	17 (34.7)	13 (25.5)	18 (36.0)
	1 year to 5 years	17 (34.7)	23 (45.1)	24 (48.0)
	>5 years	15 (30.6)	15 (29.4)	8 (16.0)
Previous treatment				
	Medication	0 (0.0)	2 (3.9)	0 (0.0)
	Oriental medicine treatment	2 (4.1)	4 (7.8)	1 (2.0)
	Therapeutic exercise	1 (2.0)	3 (5.9)	0 (0.0)
	Psychotherapy	0 (0.0)	0 (0.0)	0 (0.0)
	Other treatment	6 (12.2)	4 (7.8)	5 (10.0)
Chronic fatigue syndrome, M/F		3 (6.1)/18 (36.7)	6 (11.8)/17 (33.3)	6 (12.0)/13 (26.0)
Education >12 years		28 (57.1)	39 (76.5)	35 (70.0)
Job		36 (73.5)	39 (76.5)	40 (80.0)
Marriage		38 (77.6)	32 (62.7)	37 (74.0)
Irregular meals		18 (36.7)	18 (35.3)	19 (38.0)
Exercise				
	Yes	29 (59.2)	24 (47.1)	23 (46.0)
	Weekly average frequency	3.4 ± 2.0	2.8 ± 1.5	3.4 ± 1.6
	Average time per session, h	1.2 ± 0.9	1.4 ± 0.6	1.1 ± 0.5
Tobacco use		8 (16.3)	10 (19.6)	8 (16.0)
Alcohol use		23 (46.9)	21 (41.2)	21 (42.0)
Treatment expectancy		6.9 ± 1.5	6.1 ± 1.4	4.5 ± 2.4
	Yes (6 to 9)	40 (81.6)	37 (72.5)	18 (36.0)
	No (1 to 5)	9 (18.4)	14 (27.5)	32 (64.0)

Data are presented as either mean ± SD or number (%), where appropriate

### Findings for the primary outcome

The FSS score from Group A at 5 weeks after randomization was significantly lower than that of Group C (Table 2).

**Table 2:** Results of mixed model for repeated measures (MMRM) analysis of full analysis set (FAS)

Outcome	Group	Baseline	5 weeks			13 weeks		
		Mean (SD)	Mean (SD)	*P* ^a^	LSMD (95 % CI)^a^	Mean (SD)	*P* ^a^	LSMD (95 % CI)^a^
FSS	A	4.67 (1.27)	3.38 (1.01)	0.023	−0.43 (−0.81, −0.05)	3.34 (1.17)	0.075	−0.36 (−0.74, 0.03)
	B	4.75 (1.07)	3.58 (1.12)	0.151	−0.29 (−0.66, 0.08)	3.51 (1.19)	0.290	−0.23 (−0.62, 0.15)
	C	4.48 (1.27)	4.47 (1.18)	-	-	4.28 (1.24)	-	-
SRI-short form	A	62.56 (17.95)	42.21 (14.18)	0.032	−6.01 (−11.58, −0.44)	42.28 (14.33)	0.037	−6.19 (−12.06, −0.31)
	B	55.67 (14.40)	42.10 (13.66)	<0.001	−9.33 (−14.72, −3.95)	39.81 (13.55)	<0.001	−10.80 (−16.49, −5.12)
	C	60.04 (18.16)	56.47 (18.03)	-	-	57.41 (20.46)	-	-
BDI	A	16.41 (8.36)	9.02 (6.92)	0.252	−1.56 (−3.94, 0.83)	9.63 (6.39)	0.231	−1.68 (−4.16, 0.81)
	B	15.00 (7.67)	8.88 (7.66)	0.052	−2.34 (−4.70, 0.02)	7.89 (7.15)	0.007	−3.25 (−5.71, −0.80)
	C	15.30 (7.05)	13.36 (6.39)	-	-	14.28 (8.54)	-	-
NRS	A	6.59 (1.44)	4.42 (1.98)	<0.001	−0.92 (−1.45, −0.40)	4.77 (2.22)	0.011	−0.70 (−1.26, −0.14)
	B	6.55 (1.27)	4.52 (1.70)	<0.001	−0.90 (−1.42, −0.39)	4.51 (1.95)	0.002	−0.85 (−1.40, −0.29)
	C	6.40 (1.40)	6.53 (1.36)	-	-	6.42 (1.69)	-	-
EQ-5D	A	0.84 (0.10)	0.91 (0.11)	0.909	−0.01 (−0.04, 0.03)	0.92 (0.11)	0.844	0.01 (−0.03, 0.04)
	B	0.87 (0.09)	0.91 (0.09)	0.522	0.01 (−0.02, 0.05)	0.94 (0.08)	0.074	0.03 (0.00, 0.07)
	C	0.88 (0.11)	0.88 (0.10)	-	-	0.87 (0.15)	-	-

LSMD, least squares mean difference; CI, confidence interval; FSS, fatigue severity scale; SRI-short form, short form of the stress response inventory; BDI, Beck depression inventory; NRS, numeric rating scale; EQ-5D, EuroQol-5 dimension.

^a^ *P* value with 95 % CI, Dunnett’s adjustment

### Findings for the secondary outcomes

The FSS scores at 13 weeks after randomization were not different between the groups. The SRI-short form scores were significantly lower in the treatment groups than in the control group at both 5 and 13 weeks after randomization. Although no difference was observed in the BDI score among the groups at 5 weeks after randomization, the score from Group B was significantly reduced in comparison to Group C at 13 weeks after randomization. The NRS scores from the treatment groups were significantly reduced compared to those from the control group at both 5 and 13 weeks after randomization. The EQ-5D did not show any differences among the groups at 5 and 13 weeks after randomization (Table 2).

### Sensitivity analysis

After processing the missing values with the LOCF method, the results of the ANCOVA indicated that the scores from the FSS, SRI-short form, BDI, and NRS were significantly reduced in the treatment groups compared to the control group at both 5 and 13 weeks after randomization. The EQ-5D was significantly different between Group A and C at 5 weeks after randomization and between both treatment groups and the control group at 13 weeks after randomization (Table 3).

**Table 3:** Results of analysis of covariance (ANCOVA) of full analysis set (FAS)

Outcome	Group	Baseline	5 weeks			13 weeks		
		Mean (SD)	Mean (SD)	*P* ^a^	LSMD (95 % CI)^a^	Mean (SD)	*P* ^a^	LSMD (95 % CI)^a^
FSS	A	4.67 (1.27)	3.39 (1.12)	<0.001	−1.14 (−1.57, −0.71)	3.32 (1.17)	<0.001	−1.01 (−1.48, −0.55)
	B	4.75 (1.07)	3.62 (1.13)	<0.001	−0.95 (−1.38, −0.52)	3.58 (1.20)	<0.001	−0.80 (−1.26, −0.34)
	C	4.48 (1.27)	4.43 (1.19)	-	-	4.24 (1.25)	-	-
SRI-short form	A	62.56 (17.95)	43.07 (15.49)	<0.001	−15.24 (−21.32, −9.16)	43.61 (15.31)	<0.001	−15.01 (−21.96, −8.06)
	B	55.67 (14.40)	42.65 (14.24)	<0.001	−11.58 (−17.45, −5.72)	40.19 (13.57)	<0.001	−14.97 (−21.67, −8.26)
	C	60.04 (18.16)	56.82 (18.10)	-	-	57.36 (20.30)	-	-
BDI	A	16.41 (8.36)	9.43 (6.98)	<0.001	−5.03 (−7.60, −2.47)	10.02 (6.40)	<0.001	−4.74 (−7.56, −1.91)
	B	15.00 (7.67)	9.04 (7.61)	<0.001	−4.63 (−7.17, −2.10)	8.20 (7.01)	<0.001	−5.88 (−8.67, −3.09)
	C	15.30 (7.05)	13.84 (6.80)	-	-	14.22 (8.32)	-	-
NRS	A	6.59 (1.44)	4.56 (2.09)	<0.001	−1.96 (−2.72, −1.20)	4.78 (2.32)	<0.001	−1.64 (−2.51, −0.78)
	B	6.55 (1.27)	4.58 (1.70)	<0.001	−1.93 (−2.68, −1.18)	4.59 (1.92)	<0.001	−1.81 (−2.67, −0.96)
	C	6.40 (1.40)	6.46 (1.39)	-	-	6.34 (1.70)	-	-
EQ-5D	A	0.84 (0.10)	0.96 (0.11)	0.025	0.04 (0.00, 0.08)	0.91 (0.11)	0.008	0.06 (0.01, 0.11)
	B	0.87 (0.09)	0.91 (0.09)	0.107	0.03 (−0.01, 0.07)	0.93 (0.09)	0.005	0.06 (0.02, 0.11)
	C	0.88 (0.11)	0.88 (0.09)	-	-	0.87 (0.15)	-	-

LSMD, least squares mean difference; CI, confidence interval; FSS, fatigue severity scale; SRI-short form, short form of the stress response inventory; BDI, Beck depression inventory; NRS, numeric rating scale; EQ-5D, EuroQol-5 dimension

^a^ *P* value with 95 % CI, Dunnett’s adjustment

The results of the PPS analysis were similar to those of the FAS. However, the SRI-short form score at 13 weeks after randomization was not significantly different between Group A and C (Table 4).

**Table 4:** Results of mixed model for repeated measures (MMRM) analysis of per-protocol set (PPS)

Outcome	Group	Baseline	5 weeks			13 weeks		
		Mean (SD)	Mean (SD)	*P* ^a^	LSMD (95 % CI)^a^	Mean (SD)	*P* ^a^	LSMD (95 % CI)^a^
FSS	A	4.70 (1.22)	3.38 (1.10)	0.017	−0.45 (−0.84, −0.07)	3.34 (1.17)	0.074	−0.36 (−0.75, 0.03)
	B	4.74 (1.09)	3.58 (1.12)	0.090	−0.33 (−0.71, 0.04)	3.51 (1.19)	0.230	−0.26 (−0.64, 0.12)
	C	4.52 (1.26)	4.47 (1.18)	-	-	4.24 (1.22)	-	-
SRI-short form	A	62.59 (17.77)	42.21 (14.18)	0.039	−5.92 (−11.58, −0.27)	42.28 (14.33)	0.063	−5.69 (−11.64, 0.26)
	B	55.50 (14.23)	42.10 (13.66)	<0.001	−9.22 (−14.69, −3.76)	39.81 (13.55)	<0.001	−10.34 (−16.10, −4.58)
	C	59.26 (18.34)	56.47 (18.03)	-	-	56.67 (20.11)	-	-
BDI	A	16.59 (8.60)	9.02 (6.92)	0.338	−1.39 (−3.82, 1.04)	9.64 (6.39)	0.386	−1.35 (−3.88, 1.18)
	B	15.12 (7.67)	8.88 (7.66)	0.086	−2.14 (−4.54, 0.25)	7.89 (7.15)	0.018	−2.92 (−5.41, −0.43)
	C	14.91 (6.78)	13.36 (6.39)	-	-	14.00 (8.42)	-	-
NRS	A	6.57 (1.42)	4.42 (1.98)	<0.001	−1.01 (−1.54, −0.48)	4.77 (2.22)	0.006	−0.76 (−1.33, −0.20)
	B	6.53 (1.28)	4.52 (1.70)	<0.001	−0.98 (−1.50, −0.46)	4.51 (1.95)	<0.001	−0.90 (−1.46, −0.34)
	C	6.47 (1.38)	6.53 (1.36)	-	-	6.38 (1.69)	-	-
EQ-5D	A	0.84 (0.10)	0.91 (0.11)	0.948	0.00 (−0.04, 0.03)	0.92 (0.11)	0.785	0.01 (−0.03, 0.05)
	B	0.87 (0.10)	0.91 (0.09)	0.494	0.01 (−0.02, 0.05)	0.94 (0.08)	0.067	0.03 (0.00, 0.07)
	C	0.88 (0.11)	0.88 (0.10)	-	-	0.87 (0.15)	-	-

LSMD, least squares mean difference; CI, confidence interval; FSS, fatigue severity scale; SRI-short form, short form of the stress response inventory; BDI, Beck depression inventory; NRS, numeric rating scale; EQ-5D, EuroQol-5 dimension

^a^ *P* value with 95 % CI, Dunnett’s adjustment

The FSS score at 5 weeks after randomization, performed on the FAS after correcting for the treatment expectancy in each group, showed a significant difference between Group A and C (*P* = 0.028). A significant difference was not observed between Group B and C (*P* = 0.129).

### Subgroup analysis

The CFS group comprised 21 patients in Group A, 23 in Group B, and 19 in Group C. Furthermore, the ICF group comprised 28 patients in Group A, 28 in Group B, and 31 in Group C. Results of MMRM analysis of FSS score in each group and results of the subgroup-treatment effect interaction test are presented in Table 5.

**Table 5:** Results of the mixed model for repeated measures (MMRM) analysis of fatigue severity scale (FSS) score in the subgroups

Subgroup		Baseline	5 weeks				13 weeks			
		Mean (SD)	Mean (SD)	*P* ^a^	LSMD (95 % CI)^a^	Interaction^b^	Mean (SD)	*P* ^a^	LSMD (95 % CI)^a^	Interaction^b^
CFS (n = 63)	A	5.50 (0.94)	3.62 (1.22)	0.993	0.02 (−0.51, 0.56)	0.585^c^	3.43 (1.21)	0.924	−0.08 (−0.64, 0.48)	0.854^c^
	B	5.06 (0.87)	3.75 (1.00)	0.768	−0.14 (−0.66, 0.38)		3.95 (1.05)	0.901	−0.09 (−0.64, 0.46)	
	C	4.74 (0.81)	4.39 (0.97)	-	-		4.44 (1.23)	-	-	
ICF (n = 87)	A	4.06 (1.14)	3.21 (0.99)	0.008	−0.68 (−1.20, −0.16)		3.27 (1.15)	0.078	−0.48 (−1.01, 0.04)	
	B	4.50 (1.16)	3.44 (1.21)	0.188	−0.37 (−0.89, 0.14)		3.16 (1.20)	0.235	−0.35 (−0.87, 0.17)	
	C	4.32 (1.47)	4.51 (1.29)	-	-		4.19 (1.25)	-	-	

LSMD, least squares mean difference; CI, confidence interval; CFS, chronic fatigue syndrome; ICF, idiopathic chronic fatigue

^a^ *P* value with 95 % CI, Dunnett’s adjustment. ^b^Subgroup treatment effect interaction. ^c^ *P* value

### Concomitant treatments

Usual care methods for fatigue reported during the trial period were nutritional supplements and exercise (working out, walking, riding a stationary bicycle, swimming, and hiking). In terms of individual groups, Group A had two patients who took nutritional supplements, three patients who performed exercise, and one patient who combined nutritional supplements with exercise; whereas Group B had two patients who took nutritional supplements, one patient who received a nutritional supplement injection, and three patients who performed exercise. In Group C, two patients took nutritional supplements and three patients performed exercise. None of the participants in Group A or C received any additional acupuncture during the study period, other than the therapy provided as part of the study. One participant in Group B had received acupuncture for lower back pain, which was unrelated to fatigue.

### Adverse events

A total of 10 adverse events were reported by 10 participants. Of these events, two were determined to be linked either to acupuncture or possibly associated with acupuncture, and the symptom severity was mild in both cases. All participants who experienced adverse events improved during the research period and continued to participate in the experiment. Although two serious adverse events were reported, they were not related to the acupuncture (that is, surgery for a nasal problem and hospitalization because of a traffic accident) (Table 6).

**Table 6:** Adverse events

Symptom	Patient numbers	Start date	End date	Intensity	Causal relationship	Action related to the intervention	Treatment	Outcome
Redness and itching around CV6	1	20120918	20120920	Mild	Probably	No change	None	Cured
Right thumb numbness, pain	1	20120913	20121120	Mild	Possibly	No change	None	Improved
Nasal obstruction	1	20121031	20121031	Serious adverse event	Definitely not	No change	Medication, non-drug treatment	Cured
Left buttock pain (traffic accident)	1	20120928	20121006	Serious adverse event	Definitely not	No change	Medication	Improved
Coccyx fracture	1	20121213	Unknown	Moderate	Definitely not	No change	Medication	Improved
Right shoulder pain	1	20121105	20121130	Mild	Definitely not	No change	Medication	Improved
Left second finger pain	1	20121101	20121203	Mild	Definitely not	No change	Medication	Improved
Upper respiratory infection	3	20121123	20121201	Mild	Probably not	No change	None	Improved
		20121127	20121203	Mild	Definitely not	No change	None	Improved
		20121021	20121027	Moderate	Definitely not	No change	None	Improved

## Discussion

After 10 treatment sessions in 4 weeks, the mean FSS score in Group A improved significantly compared to Group C. This result is consistent with findings in a preliminary study on body acupuncture [12]. A previous study reported that the optimal FSS cut-off point to demarcate between fatigue and normal groups was 3.22 (sensitivity 84.1 %, specificity 85.7 %) [24]. Using this cut-off point as the reference standard, the mean post-treatment FSS score for Group A (3.38) can be considered a near-normal value. Group B demonstrated improvement after 10 treatment sessions in 4 weeks, with an FSS score of 3.58. However, the change was not statistically significant and contradicted findings in a preliminary study of Sa-am acupuncture [13]. The discrepancy between the results of the present and preliminary study may be due to the higher proportion of participants (approximately 28 %) with fatigue morbidity lasting less than 6 months in the preliminary study. The shorter duration of morbidity in the preliminary study likely eased the task of alleviating symptoms compared to the longer duration in the present study. Additionally, in the preliminary study, a sham acupuncture group was used as a control, and the Multiple Fatigue Scale (MFS) was used for assessment, neither of which was employed in this study. The MFS is a reconstituted fatigue assessment tool developed in 2000 and based on the Fatigue Assessment Inventory [30], and its correlation with the FSS has not yet been determined. These differences in the duration of symptoms, control groups, and assessment tools likely contributed to different results between this study and the preliminary study. When a significant result is required for only one of multiple primary endpoints in order to consider a trial positive, each endpoint must be tested with a significance level that has been corrected for multiplicity [31]. For the two primary endpoint tests in this study, we corrected type I errors using Dunnett’s method [32], and based on the results of the hypothesis tests, one of two null hypotheses was rejected. Therefore, the results of our trial can be considered positive.

In the auxiliary parameters, the FSS score in Group A decreased to 3.34 at 13 weeks after randomization; however, this trend was not significantly different from results in the control group. Therefore, the effects of body acupuncture therapy appears to have been short-term. Group A showed significant improvements in both the SRI-short form and NRS scores at 5 and 13 weeks after randomization. Group B showed significant improvements in the SRI-short form and NRS scores at 5 and 13 weeks after randomization, and in the BDI score at 13 weeks after randomization. Both treatment groups showed improvements in stress or depression, symptoms that are closely associated with fatigue, suggesting that body acupuncture and Sa-am acupuncture may positively influence fatigue in multidimensional or comprehensive aspects. Both Groups A and B showed a significantly lower NRS score, suggesting that body acupuncture and Sa-am acupuncture may positively influence the intensity of fatigue. Reportedly, the FSS assesses the impact rather than the intensity of fatigue [33, 34]. Thus, the NRS may measure the intensity of fatigue more efficiently than the FSS. Based on the analysis of the SRI-short form, NRS, and BDI results, it was difficult to conclude that Sa-am acupuncture had no effect to improve fatigue. Though it was beyond the scope of this study, the EQ-5D-assisted measurements of the health-related quality of life were performed as a cost-utility analysis of an economic evaluation. The results showed that neither treatment group exhibited significant improvements at 5 and 13 weeks after randomization. This might be because the treated participants did not experience any changes in their quality of life or because the EQ-5D was a generic instrument that was not disease-specific and, thus, was not sensitive enough to respond to changes in fatigue severity [35]. Similar to the primary evaluation variable analysis, the results obtained using Dunnett’s test were presented for the secondary evaluation variables. However, type I errors were not corrected for all the comparisons in this trial. All comparisons, except for the primary analysis designated before beginning the study, pose a risk of increased type I errors due to multiple tests. The results of secondary analyses do not provide answers to the research question; they should be used for obtaining useful information from the trial [31].

The natural history of chronic fatigue/CFS is still of concern: many patients reported either residual symptoms or disability at follow-up and a progression or worsening of symptoms was seen in some. [36]. Therefore, it was difficult to specify a standard for recurrent fatigue in the inclusion criteria. To date, no specific diagnostic test has been established for CFS [37]; as a result, CFS is diagnosed on the basis of exclusion, subjective clinical interpretation, and patient self-report [38]. In this trial, we included participants by relying on exclusion based on laboratory tests and case definitions. In addition, we used subjective questionnaires when evaluating the outcomes.

At the beginning of this study, we had planned to process the missing data by the LOCF method. However, since the LOCF method can present problems, such as introducing a bias from incorrect estimation of the variance in effect estimates, the main analysis method was changed to the MMRM approach, and the LOCF ANCOVA method was added as a secondary analysis. The results from the two methods were then compared, and we found that the LOCF ANCOVA underestimated the *P* value. This is consistent with the results of previous studies noting that conservative behavior of LOCF is not guaranteed. Therefore, care should be taken when using the LOCF method for analysis of clinical trial data [39, 40]. Because disease severity is different for CFS and ICF, post-hoc subgroup analysis was performed to investigate differences in the therapeutic effects in each group (CFS or ICF). The interpretation of subgroup analysis does not ascertain whether the therapeutic effects exist in each subgroup, but tries to ascertain whether the interaction between the subgroups and the therapeutic effects is significant. Since there was no interaction between the subgroups and therapeutic effects in the present study, acupuncture appears to have been equally effective in all the subgroups. However, because this was an exploratory subgroup analysis, the reproducibility needs to be confirmed in other trials [41].

Although there are no traditional Chinese medical terms that describe CFS and ICF, the concepts may be similar to consumptive disease (a general term for chronic deficiency diseases due to consumption of *yin*, *yang*, *qi*, and blood) or fatigue due to overexertion (a diseased state caused by overexertion, manifested as fatigue, lassitude, shortness of breath upon exertion, and spontaneous sweating) [14, 42]. It was reported that the visceral pattern identification of CFS/ICF can be classified into spleen-lung *qi* deficiency, spleen-kidney *yang* deficiency, heart-spleen blood deficiency, and liver-kidney *yin* deficiency [42, 43]. However, we did not apply pattern identification because we could not find a tool that was sufficiently validated.

In this trial, the acupoints used in Group A were selected to relieve the mind, clear the spirit, tranquilize the body to free it from tension, and regulate the functions of the internal organs [12]. Sa-am acupuncture is largely divided into fixed pattern, transformed pattern, and experienced prescription. Transformed pattern (LU8, SP3, HT8) and experienced prescription (BL15, CV6) were applied to the participants in Group B [44, 45]. In TCM, needle therapy harmonizes the *qi* that flows through the meridians of the body. Harmonizing the *qi* is effective when the *qi* is obtained in response to acupuncture needling (*de qi*, needling sensation). Among the methods for harmonizing the *qi*, *cui qi* or hastening the *qi*, is a way of facilitating *de qi* if *qi* does not come when it should, whereas *xing qi* or moving the *qi*, can aid in transferring the needling response to the disease location. *Bu xie*, or supplementation and draining, is a method of reinforcing what is lacking and reducing what is excessive. Manual acupuncture can be categorized as basic, assistant, *bu xie*, as well as comprehensive *bu xie* manual. The basic manual method is a simple technique that involves induction of *cui qi* and *xing qi*, as well as *bu xie*. This technique includes twirling and needle retention. Twirling involves turning the needle to the left and to the right, whereas needle retention is designed to maintain the needle at a specific point for a period of time. These basic manual methods can be applied as basic *bu xie* manual acupuncture techniques. Among the basic *bu xie* manual methods, the directional method includes ‘*bu*’, which is supplementation via insertion of the needle in the direction of the needled channel flow and ‘*xie*’, which is draining via insertion of the needle in the direction opposite to the channel flow. The nine-six method, which is another basic *bu xie* manual method, distinguishes the number of movements involved in twirling or needle retention, where the number 9 represents ‘*bu*’ and 6 represents ‘*xie*’ [14, 46, 47]. If one locates the point accurately, *qi* may be obtained immediately after one inserts the needle to the right depth in the right direction [47]. In the present study, needling was performed in Group A by obtaining accurate acupoints and a suitable depth of needling, along with proper perception by the acupuncturist. Therefore, although twirling and *bu xie* were not performed in Group A, it is believed that the *qi* was correctly obtained. In the original literature on Sa-am acupuncture, detailed descriptions of *bu xie* were not provided, presumably because it was a general contemporary *bu xie* method or because it was a method based on the traditional *bu xie* method from Neijing [48]. In Group B, needling was performed by the manual methods (twirling, needle retention, and directional methods) used in the preliminary study [13], as well as the nine-six method added in this trial.

This study has three primary strengths. First, the external validity of the study was improved by performing a pragmatic clinical trial (PCT). Clinical trials can have either an explanatory or a pragmatic design. An explanatory clinical trial (ECT) evaluates what interventions work in ideal (controlled) situations, whereas a PCT evaluates whether interventions work in everyday clinical practice settings. Since PCTs reflect clinical practice, the patient population range is broader than in ECTs, and PCTs often allow for individualized treatments for patients [21, 22]. In previous studies, acupuncture therapy for chronic fatigue has often been limited to CFS [7–11]. In this study, we used usual care as the control and we included ICF patients, as well as CFS patients, in the subject population. Moreover, the study was intended to better reflect real clinical situations through its multi-institutional clinical trial design. However, we did not evaluate the long-term effects of the treatment investigated, unlike general PCTs. In addition, we used a standardized treatment and have therefore studied the effectiveness of the selected points. Second, the results from the FAS and PPS were presented together. A FAS is the collection of participants who most closely fit the ITT principle of the clinical trial, and which minimizes the number of participants excluded from analysis. A PPS is a subset of the FAS and consists of fully compliant participants. In this study, since the results for the two sets were similar, confidence in the trial outcomes was increased [28]. Third, adverse events were reported in detail. We described the symptoms and signs, dates of presentation and disappearance, causal relationships with test interventions, acupuncture-related actions, treatments, and results. During the clinical trial period, 2 of the 150 participants (1.3 %) showed acupuncture-related adverse events. The safety of acupuncture therapy has been reported previously [49–51] and is confirmed herein.

This study has some weaknesses. First, the effectiveness of acupuncture in this study included both specific effects and context effects (expectation, patient-practitioner relationship, etc.) [52]. Although the results compensated for treatment expectations, which are presented in the results section as a reference, the specific effects of acupuncture could not be isolated owing to the study design. It was reported that the identities of CFS patients are challenged when the legitimacy of their illness is questioned, and doctors can support patients in coping with their disease by encouraging them instead of casting suspicion upon them [53]. In the present study, free communication between the patients and the acupuncturist was allowed. However, acupuncturists did not actively provide attention, advice, or empathy. Therefore, it is thought that the patient-practitioner relationship did not significantly impact the treatment effects. Second, there is lower internal validity owing to the allowance of individualized usual care (except acupuncture therapy for chronic fatigue). Although the percentage of participants who reported using usual care during the trial period was not high (Group A: 12 %; B: 12 %; and C: 10 %), its effect on the study’s outcomes could not be evaluated. Third, fatigue could not be evaluated from multidimensional aspects. The FSS is one of the best known and most used fatigue scales [33, 54]. Since the reliability and validity of the Korean-language version of the FSS has been proven [24], it was used as the primary evaluation instrument for this study. Although the FSS consists of multiple items, it is still a one-dimensional scale and, therefore, is not sufficient for the evaluation of complex aspects of fatigue such as its physical, psychological, and social dimension features [55]. However, there is no instrument among the multidimensional fatigue scales that is used globally and that has been translated into Korean and tested for reliability and validity. In consequence, we did not use a multidimensional instrument.

Although this study did not conclusively demonstrate the effectiveness of acupuncture for CFS and ICF, the results could serve as a reference in the treatment of patients with chronic fatigue of unknown causes.

## Conclusions

The results of this study suggest that 4 weeks of body acupuncture therapy added to usual care can improve fatigue symptoms of CFS and ICF patients.

## Additional files

Additional file 1:
**Flow diagram, Study flow diagram.** (DOCX 43 kb) Additional file 2:
**CONSORT (Consolidated Standards of Reporting Trials) checklist, CONSORT 2010 checklist with the non-pharmacological trials extension to CONSORT.** (DOCX 26 kb)

## Abbreviations

ALT: alanine aminotransferase
ANCOVA: analysis of covariance
AST: aspartate aminotransferase
BDI: Beck Depression Inventory
BMI: body mass index
BP: blood pressure
CDC: Centers for Disease Control and Prevention
CFS: chronic fatigue syndrome
CRIS: Clinical Research Information Service
CXR: chest radiography
ECG: electrocardiography
ECT: explanatory clinical trial
EQ-5D: EuroQol-5 Dimension
FAS: full analysis set
FSS: Fatigue Severity Scale
FT4: free thyroxine
Hb: hemoglobin
hCG: human chorionic gonadotropin
Hct: hematocrit
ICF: idiopathic chronic fatigue
ITT: intention to treat
LOCF: last observation carried forward
MFS: Multiple Fatigue Scale
MMRM: mixed model for repeated measures
NRS: Numeric Rating Scale
PCT: pragmatic clinical trial
PPS: per-protocol set
SAS: Strategic Applications Software
SRI: Stress Response Inventory
TCM: traditional Chinese medicine
TFT: fhyroid function test
TSH: thyroid stimulating hormone
VAS: Visual Analogue Scale
WBC: white blood cells
