# Supplementary material for: Acupuncture for chronic fatigue syndrome and idiopathic chronic fatigue: a multicenter, nonblinded, randomized controlled trial
Source: Trials. 2015 Jul 26;16:314. doi: 10.1186/s13063-015-0857-0 (PMC4515016; doi:10.1186/s13063-015-0857-0)
Supplement: Additional file 1: — Flow diagram, Study flow diagram. (DOCX 43 kb) [file 13063_2015_857_MOESM1_ESM.docx]

Assessed for eligibility (n=195)

Randomized (n=150)

Excluded (n=45)

- Not meeting inclusion criteria (n=39)

- Declined to participate (n=6)

- Other reasons (n=0)

Allocated to Group A (body acupuncture plus usual care, n=49)

- Received allocated intervention (n=49)

- Did not receive allocated intervention (n=0)

Allocated to Group B (Sa-am acupuncture plus usual care, n=51)

- Received allocated intervention (n=51)

- Did not receive allocated intervention (n=0)

Allocated to Group C (usual care alone, n=50)

- Stayed on control (n=50)

- Did not stay on control (n=0)

Lost to follow-up (n=5)

- Dropped out of the intervention (n=3)

- Did not complete measurement (n=2)

Discontinued intervention (n=3)

- Withdrawal of consent (n=2)

- Poor compliance (n=1)

Lost to follow-up (n=4)

- Dropped out of the intervention (n=2)

- Did not complete measurement (n=2)

Discontinued intervention (n=2)

- Withdrawal of consent (n=1)

- Lost contact (n=1)

Lost to follow-up (n=2)

- Dropped out (n=2)

Discontinued (n=2)

- Withdrawal of consent (n=2)

Analysed (n=49)

- Excluded from analysis (n=0)

Analysed (n=51)

- Excluded from analysis (n=0)

Analysed (n=50)

- Excluded from analysis (n=0)

## Allocation

## Enrollment

## Analysis

## Follow-Up
