# Supplementary material for: Acupuncture for chronic fatigue syndrome and idiopathic chronic fatigue: a multicenter, nonblinded, randomized controlled trial
Source: Trials. 2015 Jul 26;16:314. doi: 10.1186/s13063-015-0857-0 (PMC4515016; doi:10.1186/s13063-015-0857-0)
Supplement: Additional file 2: — CONSORT (Consolidated Standards of Reporting Trials) checklist, CONSORT 2010 checklist with the non-pharmacological trials extension to CONSORT. (DOCX 26 kb) [file 13063_2015_857_MOESM2_ESM.docx]

***Citation:*** *MacPherson H, Altman DG, Hammerschlag R, Youping L, Taixiang W, White A, Moher D; STRICTA Revision Group. Revised STandards for Reporting Interventions in Clinical Trials of Acupuncture (STRICTA): Extending the CONSORT statement.* *J Evid Based Med. 2010;3(3):140-55.*

**Table 1** STRICTA 2010 checklist of information to include when reporting interventions in a clinical trial of acupuncture

| **Item** | **Detail** | **Section** |
| --- | --- | --- |
| **1. Acupuncture rationale** | (a) Style of acupuncture (e.g. Traditional Chinese Medicine, Japanese, Korean, Western medical, Five Element, ear acupuncture, etc) | “Interventions”, “Group A”, and “Group B” |
|  | (b) Reasoning for treatment provided, based on historical context, literature sources, and/or consensus methods, with references where appropriate |  |
|  | (c) Extent to which treatment was varied |  |
| **2. Details of needling** | (a) Number of needle insertions per subject per session (mean and range where relevant) | “Interventions”, “Group A”, and “Group B” |
|  | (b) Names (or location if no standard name) of points used (uni/bilateral) |  |
|  | (c) Depth of insertion, based on a specified unit of measurement, or on a particular tissue level |  |
|  | (d) Response sought (e.g. *de qi* or muscle twitch response) |  |
|  | (e) Needle stimulation (e.g. manual, electrical) |  |
|  | (f) Needle retention time |  |
|  | (g) Needle type (diameter, length, and manufacturer or material) |  |
| **3. Treatment regimen** | (a) Number of treatment sessions | “Interventions” |
|  | (b) Frequency and duration of treatment sessions |  |
| **4. Other components of treatment** | (a) Details of other interventions administered to the acupuncture group (e.g. moxibustion, cupping, herbs, exercises, lifestyle advice) | “Concomitant treatments” |
|  | (b) Setting and context of treatment, including instructions to practitioners, and information and explanations to patients | “Interventions” |
| **5. Practitioner background** | Description of participating acupuncturists (qualification or professional affiliation, years in acupuncture practice, other relevant experience) | “Interventions” |
| **6. Control or comparator interventions** | (a) Rationale for the control or comparator in the context of the research question, with sources that justify this choice | “Group C” and “Concomitant treatments” |
|  | (b) Precise description of the control or comparator. If sham acupuncture or any other type of acupuncture-like control is used, provide details as for Items 1 to 3 above. |  |

Note: This checklist, which should be read in conjunction with the explanations of the STRICTA items provided in the main text, is designed to replace CONSORT 2010’s item 5 when reporting an acupuncture trial.

**Table 2** CONSORT 2010 checklist with the Non-pharmacological Trials Extension to CONSORT (with STRICTA 2010 extending CONSORT Item 5 for acupuncture trials)

| **Section/Topic** | **Item #** | **CONSORT 2010 Statement: checklist item. Describe:** | **Additional items from the non-pharmacological trials extension to CONSORT. Add:** | **Section** |
| --- | --- | --- | --- | --- |
| *TITLE AND ABSTRACT* |  |  |  |  |
|  | 1a | Identification as a randomized trial in the title | In the abstract, description of the experimental treatment, comparator, care providers, centres and blinding status. | “Title” |
|  | 1b | Structured summary of trial design, methods, results, and conclusions; for specific guidance see CONSORT for Abstracts |  | “Abstracts” |
| *INTRODUCTION* |  |  |  |  |
| Background and objectives | 2a | Scientific background and explanation of rationale |  | “Background” |
|  | 2b | Specific objectives or hypotheses |  | “Background” |
| METHODS |  |  |  |  |
| Trial design | 3a | Description of trial design (e.g., parallel, factorial) including allocation ratio |  | “Methods” |
|  | 3b | Important changes to methods after trial commencement (e.g. eligibility criteria), with reasons |  | Not applicable |
| Participants | 4a | Eligibility criteria for participants | When applicable, eligibility criteria for centres and those performing the interventions. | “Methods” |
|  | 4b | Settings and locations where the data were collected |  | “Methods” |
| Interventions | 5 | The interventions for each group with sufficient details to allow replication, including how and when they were actually administered | Precise details of both the experimental treatment and comparator - see Table 1 for details | Table 1 (STRICTA checklist) |
| Outcomes | 6a | Completely defined pre-specified primary and secondary outcome measures, including how and when they were assessed |  | “Primary outcome” and “Secondary outcomes” |
|  | 6b | Any changes to trial outcomes after the trial commenced with reasons |  | Not applicable |
| Sample size | 7a | How sample size was determined | When applicable, details of whether and how the clustering by care providers or centres was addressed. | “Statistical analysis” |
|  | 7b | When applicable, explanation of any interim analyses and stopping guidelines |  | Not applicable |
| Randomization |  |  |  |  |
| *Sequence generation* | 8a | Method used to generate the random allocation sequence | When applicable, how care providers were allocated to each trial group. | “Randomization” |
|  | 8b | Type of randomization; details of any restriction (e.g., blocking and block size) |  | “Randomization” |
| *Allocation concealment* | 9 | Mechanism used to implement the random allocation sequence (e.g., sequentially numbered containers), describing any steps taken to conceal the sequence until interventions were assigned |  | “Randomization” |
| *Implementation* | 10 | Who generated the random allocation sequence, who enrolled participants, and who assigned participants to interventions |  | “Randomization” |
| Blinding | 11a | If done, who was blinded after assignment to interventions (e.g. participants, care providers, those assessing outcomes) and how | Whether or not those administering co-interventions were blinded to group assignment. If blinded, method of blinding and description of the similarity of interventions. | “Blinding” |
|  | 11b | If relevant, description of the similarity of interventions |  | Not applicable |
| Statistical methods | 12a | Statistical methods used to compare groups for primary and secondary outcomes | When applicable, details of whether and how the clustering by care providers or centres was addressed. | “Statistical analysis” |
|  | 12b | Methods for additional analyses, such as subgroup analyses and adjusted analyses |  | “Statistical analysis” and “Table 2–5” |
| RESULTS |  |  |  |  |
| Participant flow (A diagram is strongly recommended) | 13a | For each group, the numbers of participants who were randomly assigned, received intended treatment, and were analyzed for the primary outcome | The number of care providers or centres performing the intervention in each group and the number of patients treated by each care provider or in each centre. | “Flow of participants” and “Additional file 1” |
|  | 13b | For each group, losses and exclusions after randomization, together with reasons |  | “Additional file 1” |
| Implementation of intervention |  |  | Details of the experimental treatment and comparator as they were implemented. | “Additional file 1” |
| Recruitment | 14a | Dates defining the periods of recruitment and follow-up |  | “Methods” and “Additional file 1” |
|  | 14b | Why the trial ended or was stopped |  | Not applicable |
| Baseline data | 15 | A table showing baseline demographic and clinical characteristics for each group | When applicable, a description of care providers (case volume, qualification, expertise, etc.) and centres (volume) in each group. | “Baseline characteristics” and “Table 1” |
| Numbers analyzed | 16 | For each group, number of participants (denominator) included in each analysis and whether the analysis was by original assigned groups |  | “Additional file 1” |
| Outcomes and estimation | 17a | For each primary and secondary outcome, results for each group, and the estimated effect size and its precision (e.g., 95% confidence interval) |  | “Findings for the primary outcome”, “Findings for the secondary outcomes”, and “Table 2” |
|  | 17b | For binary outcomes, presentation of both absolute and relative effect sizes is recommended |  | Not applicable |
| Ancillary analyses | 18 | Results of any other analyses performed, including subgroup analyses and adjusted analyses, distinguishing pre-specified from exploratory |  | “Sensitivity analysis”, “Subgroup analysis”, and “Table 3–5” |
| Harms | 19 | All important harms or unintended effects in each group; for specific guidance see CONSORT for Harms |  | “Adverse events” and “Table 6” |
| DISCUSSION |  |  |  |  |
| Limitations | 20 | Trial limitations, addressing sources of potential bias, imprecision, and, if relevant, multiplicity of analyses |  | “Discussion” |
| Generalizability | 21 | Generalizability (external validity, applicability) of the trial findings | Generalizability (external validity) of the trial findings according to the intervention, comparators, patients and care providers and centres involved in the trial. | “Discussion” |
| Interpretation | 22 | Interpretation consistent with results, balancing benefits and harms, and considering other relevant evidence | In addition, take into account the choice of the comparator, lack of or partial blinding, unequal expertise of care providers or centres in each group. | “Discussion” |
| *Other Information* |  |  |  |  |
| Registration | 23 | Registration number and name of trial registry |  | After abstract |
| Protocol | 24 | Where the full trial protocol can be accessed, if available |  | Not applicable |
| Funding | 25 | Sources of funding and other support (e.g., supply of drugs); role of funders |  | “Sources of funding” |
